# Terminal deoxynucleotidyl transferase: Properties and applications

**DOI:** 10.1016/j.engmic.2024.100179

**Published:** 2024-11-28

**Authors:** Chengjie Zhang, Hizar Subthain, Fei Guo, Peng Fang, Shanmin Zheng, Mengzhe Shen, Xianger Jiang, Zhengquan Gao, Chunxiao Meng, Shengying Li, Lei Du

**Affiliations:** a State Key Laboratory of Microbial Technology, Shandong University, Qingda 266237, Shandong, China; b School of Pharmacy, Binzhou Medical University, Yantai 264003, Shandong, China; c BGI Research, Shenzhen 518083, China; d Laboratory for Marine Biology and Biotechnology, Qingdao National Laboratory for Marine Science and Technology, Qingdao 266237, Shandong, China

**Keywords:** Terminal deoxynucleotidyl transferase, Template-independent DNA synthesis, Synthetic biology, Enzyme engineering

## Abstract

Terminal deoxynucleotidyl transferase (TdT), a unique DNA polymerase, can elongate DNA by adding deoxynucleotides to the 3′ terminal of a DNA chain in a template-independent manner. Owing to their remarkable DNA synthesis activity, TdTs have promoted the development of numerous nucleic acid-based methods, tools, and associated applications, attracting broad interest from both academia and industry. This review summarizes and discusses the recent research on TdTs, including their biochemical characteristics, enzyme engineering, and practical applications. New insights and perspectives on the future development of TdTs are provided.

## Graphical abstract

**Figure:**
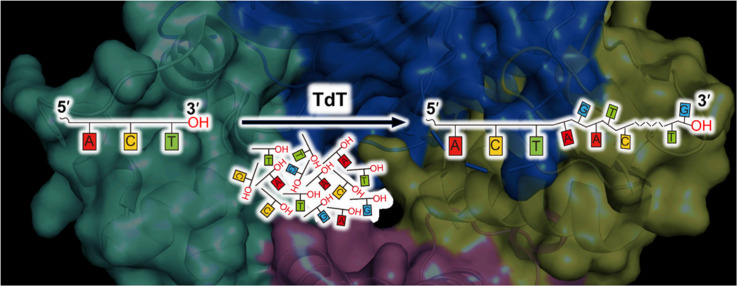

## 1 Introduction

The first terminal deoxynucleotidyl transferase (TdT) was discovered by Bollum in the thymus glands of calves in 1960 [[1], [2], [3]]. Biologically, this unique class of DNA polymerases plays a key role in the immunological responses of animals to resist infections [4]. TdTs can indiscriminately add deoxynucleotide triphosphates (dNTPs) to the 3′‑hydroxyl group (3′-OH) of single and double-stranded DNA molecules, leading to DNA elongation in a template-independent manner [3,5]. This unusual biochemical function has attracted considerable attention for some time [6]. Because of their unique catalytic properties, promising technologies for enzyme-based DNA synthesis have been developed in different fields [[7], [8], [9]].

TdT shares several common characteristics (such as sequence homology, organization of polymerase domains, and catalytic mechanism) with other proteins in the DNA polymerase X family (DNA polymerases β, λ, μ, and TdT); however, it differs in key structural elements/domains and enzymatic properties [10,11]. The structural biology and associated enzyme engineering of TdTs have not only provided significant insights into the molecular mechanism of nucleotide incorporation [12], but have also enabled the development of better TdT mutants for extended applications. To date, TdTs have been applied in various nucleotide-related areas, such as DNA synthesis [[13], [14], [15]], biosensors [[16], [17], [18]], medical diagnostics [19,20], and data storage [21].

This review summarizes the current knowledge of TdTs with respect to their catalytic properties, biological functions, enzyme engineering, and other applications. We also provided new insights and perspectives for future studies on TdTs. In the era of synthetic biology, an increasing number of research and development efforts will depend on DNA manipulation technologies, such as the synthesis of long-chain DNA molecules and the assembly of multiple genes and even whole chromosomes. Therefore, TdTs are expected to play an increasingly important role in biotechnology.

## 2 Structure, evolution, function, and activity of TdT

### 2.1 Structural features of TdT

**Fig. 1:**
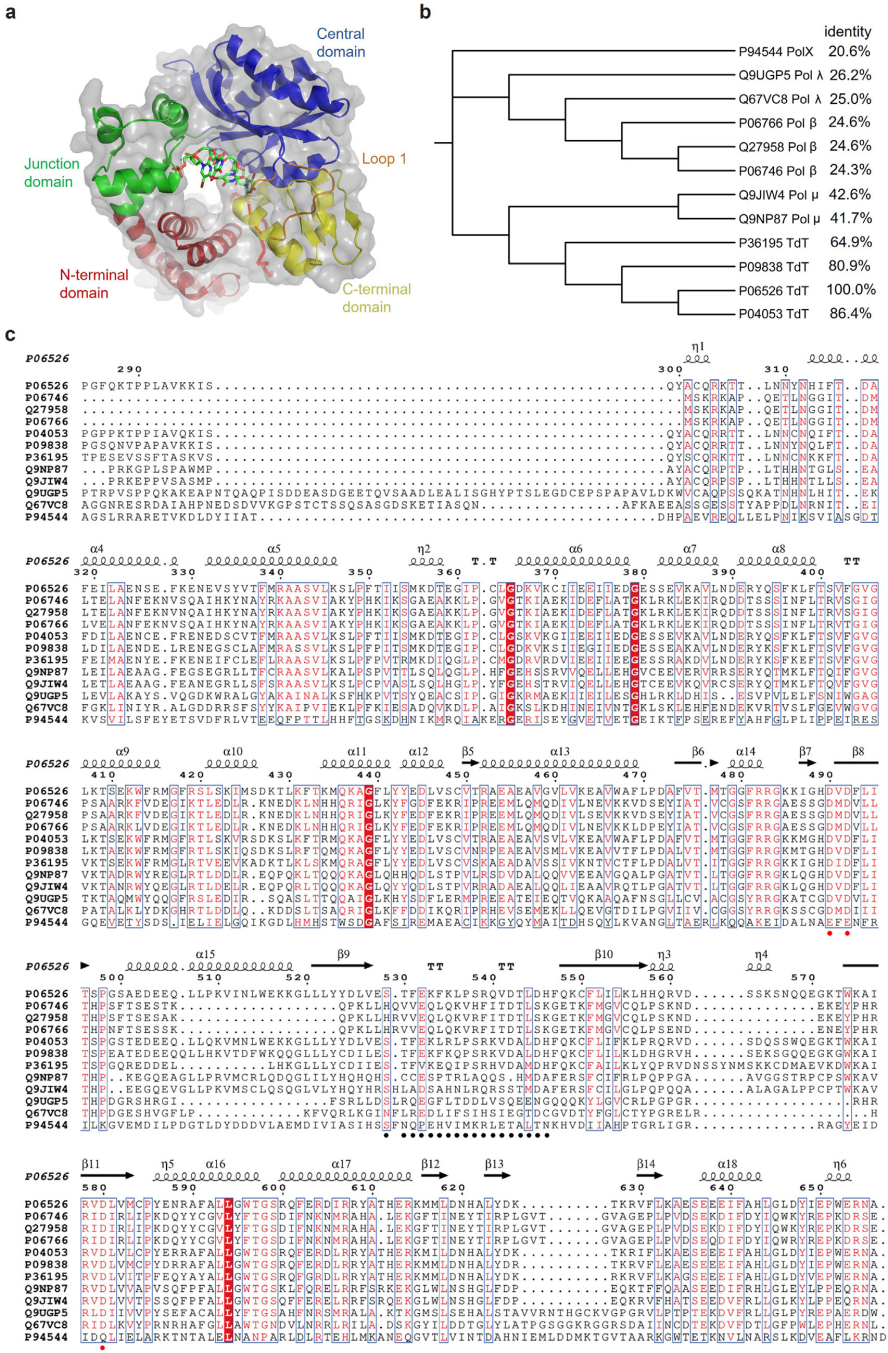
Crystal structure of the catalytic core of *Mus musculus* TdT and phylogenetic analysis of pol X family. (a) The TdT catalytic core, with the *N*-terminal BRCT domain truncated, consisting of the *α*-helical *N*-terminal, *α*-helical junction, central, and *C*-terminal subdomains. These four subdomains correspond to the 8 kDa, finger, palm, and thumb domains in pol β, respectively. (b) Phylogenetic analysis of DNA polymerase X family proteins. (c) Multiple sequence alignment of polymerase X family proteins, with the three catalytic aspartate residues marked by solid red circles and loop 1 indicated by black circles.

The first crystal structure of the catalytic core of TdT (C-TdT) from *Mus musculus* was resolved at a 2.35-Å resolution in 2022, revealing a ring-like architecture with four structural domains: an *α*-helical *N*-terminal domain (amino acids 163–243), an *α*-helical junction domain (amino acids 243–302), a central domain (amino acids 302–450), and a *C*-terminal domain (amino acids 450–510) (Fig. 1a) [22]. These domains resemble a right-hand structure and are homologous to the 8 kDa, finger, palm, and thumb domains, respectively, in DNA polymerase β (pol β), another member of the DNA polymerase X family [22,23]. Subsequently, different states of high-resolution TdT crystal structures have been reported, including pre-, post-catalytic, and competent states, facilitating our understanding of the catalytic cycle and relevant mechanisms of TdTs [12,24].

Two important conserved motifs (ALLGW(T/S)GSR and TGGFRRG) are located at the interface of the TdT catalytic center and *C*-terminal domain, containing characteristic *cis*-peptide bonds [25]. The former motif is involved in the open-closed conformational transition, whereas the latter contains an arginine residue critical for TdT catalytic activity. Structural analysis indicated that TdT adopts a two-metal ion mechanism for nucleotide transfer, similar to that of the pol I (Klenow-like folding type) superfamily. The two metal ion sites, namely metal A and metal B, are involved in activating the 3′-OH group of the primer strand and binding nucleotides [26]. The effects of different divalent metal ions, such as Mn^2+^, Co^2+^, and Zn^2+^, on the two sites have already been examined [24]. The presence of Zn^2+^ or Co^2+^ at the metal A site increases the affinity of TdT for the primer strand by inducing conformational changes in the primer strand within the ternary structure. Metal A in TdT is temporary because it exists during the binding step and leaves after this step [12]. The conformation of the sugar on the 3′ terminus of the primer strand can also affect the catalytic efficiency of TdT. In the presence of Mn^2+^ or Mg^2+^, the sugar changes from a C2′-endo to a C3′-endo conformation to facilitate the attack of the free 3′-OH group of the incoming nucleotide [12].

### 2.2 Evolutionary analysis of TdT

Based on sequence similarity and phylogenetic analysis, TdTs belong to the X family of DNA polymerases (pol X and subfamilies pol λ, pol β, pol μ, and TdT) (Fig. 1b). They belong to a subclass of the nucleotidyl transferase superfamily [[27], [28], [29], [30]]. Pol X polymerases are distributed across all organism domains, including bacteria, viruses, and eukaryotic cells. They are primarily involved in DNA repair rather than replication. Eukaryotic Pol Xs play a role in double-stranded break repair and base excision repair. Pol λ, pol β, and pol μ exhibit deoxyribose phosphate (dRP) lyase activity, while TdT is characterized by template-independent polymerase and end-bridging activities [[31], [32], [33], [34]]. Prokaryotic Pol Xs typically possess gap-filling functions, including dRP lyase and 3′−5′ exonuclease activities [35,36]. Most DNA polymerases contain conserved finger, palm, and thumb subdomains, which are crucial for nucleotide recognition/binding, polymerase catalytic activity, and DNA substrate binding, respectively [37]. Additionally, the *N*-terminal 8 kDa subdomain, which is involved in 5′-dRP-lyase function, is conserved across both eukaryotic and prokaryotic PolXs. The BRCT subdomain in eukaryotic TdT, pol λ, and pol μ is associated with protein interactions during non-homologous end-joining (NHEJ) and V(D)J recombination.

The phylogenetic tree reveals that TdT has a closer evolutionary relationship with pol μ, sharing more than 40% sequence similarity, compared to the less than 30% similarity with pol λ, pol β, and bacterial pol X (Fig. 1b). A key structural difference between TdT and template-dependent polymerases is the presence of the long lariat-like Loop1, which sterically blocks the access of a potential template strand to the catalytic site [38] (Fig. 1c). Grafting this loop onto pol μ can confer TdT-like activity, highlighting its importance in TdT function [38]. Interestingly, terminal deoxynucleotidyl transferase activity is not unique to TdT. Other members of the pol X family also exhibit template-independent activity. Some members of this family, such as the invertebrate PolX from *Ramazzottius varieornatus*, display unique traits, such as higher selectivity for dATP and dTTP over dGTP and dCTP [39].

### 2.3 Biological function of TdT

**Fig. 2:**
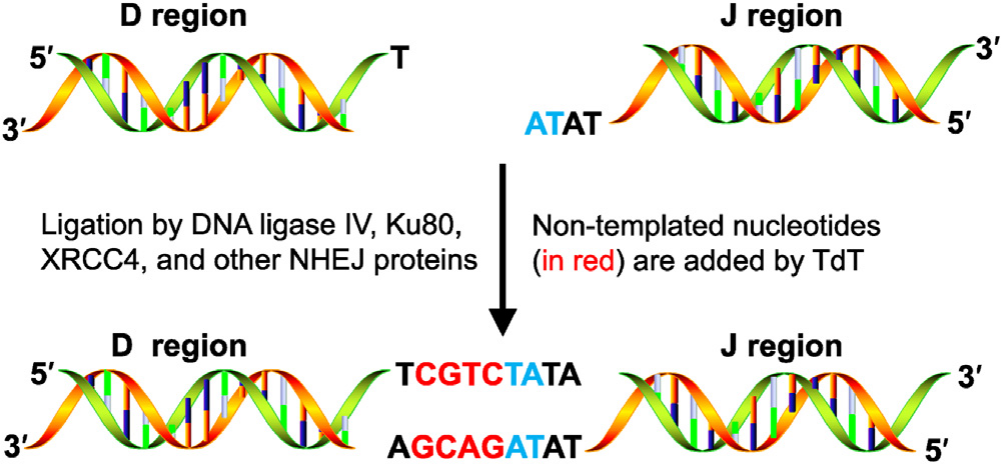
Biological functions of TdT. TdT is involved in the imprecise coding joint of V(D)J recombination, which is responsible for the diversification of immunoglobulin (Ig) and T-cell receptor genes. Firstly, TdT randomly adds several nucleotides at the 3′ termini of coding ends. Subsequently, other enzymes, such as non-homologous end-joining proteins, facilitate recombination to form complete coding genes.

The earliest hypotheses regarding the biological functions of TdT date back to the early 1970s. TdT acts as a mutagen and participates in the formation of diverse immunoglobulin (Ig) and T-cell receptor genes [19,40], as these gene rearrangements occur in prolymphocytes that express TdT [41]. In the V(D)J recombination process, which is responsible for the diversification of antibody and lymphocyte repertoires [[42], [43], [44]], TdT is expressed in pre-B and pre-T lymphocytes and contributes greatly to the diversity of the immune repertoire by randomly adding ∼1–10 nucleotides at the coding ends (non-templated nucleotides) (Fig. 2) [6,42,[45], [46], [47], [48], [49], [50]]. Notably, TdT does not add random nucleotides during V(D)J recombination independently (random collision with DNA ends) but with the assistance of other NHEJ DNA repair pathway enzymes, such as KU80 and XRCC4 [51,52], to be recruited to the V(D)J recombinase complex [4,45]. The BRCA1 *C*-terminal (BRCT) domain in the *N*-terminal region of the TdT plays a crucial role in protein recruitment for DNA repair [53]. In addition, TdT may be affected by Ku80 expression, nuclear localization, and enzyme catalysis [45].

In several species, such as mice, humans, and cattle, two splice variants of TdTs have been identified: a short form (TdTS) and a long form (TdTL, including the TdTL1 and TdTL2 types) [[54], [55], [56], [57]]. Generally, the long isoform of TdT possesses 3′→5′ exonuclease activity and can catalyze the deletion of nucleotides at hairpin coding ends but not signal ends of V(D)J joins. The short isoform of TdT produces *N* region addition at the coding ends during V(D)J recombination. TdT splice variants play crucial roles in regulating the length and composition of gene segments in V(D)J joints, contributing to the diversification of lymphocyte repertoires [58,59].

### 2.4 Biochemical activity of TdT

**Fig. 3:**
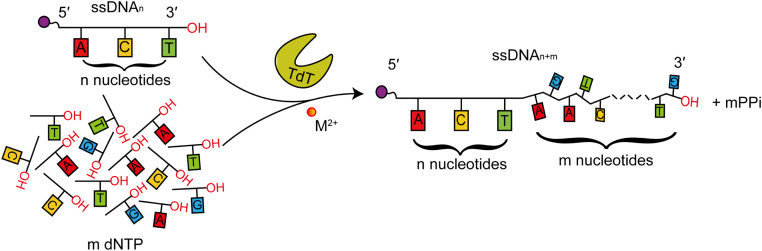
The enzymatic reaction catalyzed by TdT. TdT mediates the random addition of dNTPs to the 3′‑hydroxyl group of single-stranded DNA, generating the byproduct PPi. M^2+^ represents a divalent metal ion.

TdT catalyzes the elongation of single-stranded DNA (ssDNA) by adding dNTPs to the 3′-end of a primer, facilitated by metal ions (Fig. 3). This reaction is irreversible because of the lack of exchange between free pyrophosphate and nucleoside triphosphate, of which elongation is the primary controlling factor [60,61]. The enzymatic activity of TdT is influenced by many factors, including substrate initiators, extensors, metal ions, active-site substitutions, and buffers [[62], [63], [64]].

**Fig. 4:**
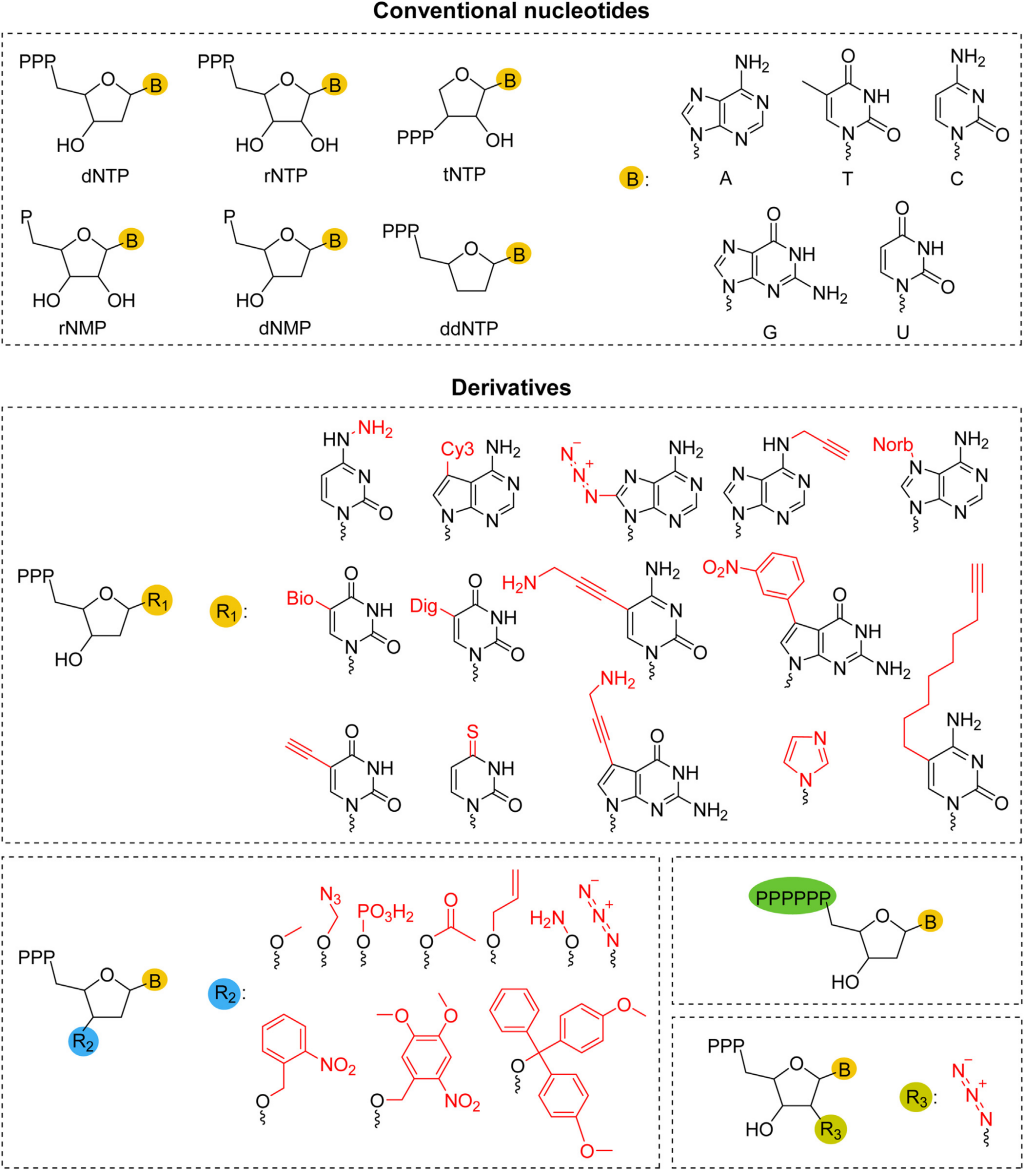
Monomer substrates of TdT. TdT can catalyze natural substrates, such as dNTPs, as well as their derivatives, including base-, sugar-, and phosphate-modified variants, and their analogs. Cy3: cyanine 3. Norb: norbornene. Bio: biotin. Dig: digoxigenin.

Initiators are essential for TdT enzymatic reactions. TdT can recognize initiators of at least three nucleotides (*i.e.*, d(pNpNpN)), but not diphosphate forms (*i.e.*, d(NpNpN)) [3,22,65]. Under specific circumstances, such as being linked to relatively long hexaethylene glycol strands that can act as initiator strands, monomer and dimer nucleotides can also be extended [66]. Both conventional and modified nucleotides (Fig. 4), such as Cy3-dNTP, 3′-*O*-azidomethyl dNTP, fluorescent-dNTP, digoxigenin/biotin-dNTP, or threose nucleic acid (TNA, a kind of xeno-nucleic acid) triphosphate (tNTP) blocks, can be used as extension units [[67], [68], [69], [70], [71], [72], [73], [74]]. In addition, rNTP, rNMP, or dNMP can be added by TdT to double-stranded DNA or RNA, serving as alternative primer substrate [[75], [76], [77]].

Metal ions are important ligands that significantly affect the enzymatic activity of TdT [75]. Divalent metal ions have different effects on the substrate selectivity and elongation properties of TdT. Specifically, Co^2+^ leads to the production of longer products when dA, dT, and dC tails [78]. Zn^2+^ can enhance the reaction rate in the presence of Mg^2+^ and affect the affinity of the TdT for initiators and dNTPs [79]. Moreover, Co^2+^ confers the highest catalytic activity for engineered TdTs among Co^2+^, Mn^2+^, Zn^2+^, and Mg^2+^ when using 3′-blocked dNTPs, such as 3′-ONH_2_-dNTPs [80].

The reaction buffer system can also influence the enzymatic catalytic activity of TdT. Tris acetate and potassium acetate are commonly used to preserve normal TdT [81]. A sulfhydryl reagent such as cysteine or 2-mercaptoethanol is beneficial for maintaining a rapid incorporation rate [82], whereas pyrophosphate and higher ionic strength buffers can inhibit the synthesis rate of TdT [62,83]. The relative effectiveness of several buffers, including cacodylate, 2-(*N*-morpholino)-ethanesulfonic acid, and *N*-2-hydroxyethylpiperazine-*N′*-2-ethanesulfonic acid has also been studied, showing a certain degree of improvement in the polymerization rate [84].

### 2.5 Optimization of the catalytic activity of TdT

These special biochemical function of TdT, which enables the elongation of DNA strands without a template, have great potential for applications in the field of polynucleotide synthesis. However, the catalytic rate and thermostability of wild-type TdTs are suboptimal, and the original TdTs cannot efficiently incorporate 3′-modified nucleotides [13,85]. Various enzyme engineering strategies have been used to overcome these limitations and enhance the performance of TdTs, and considerable progress has been made in recent years.

One of the early challenges in TdT engineering was to increase its activity and production. Roychoudhury et al. found that replacing Mg^2+^ with Co^2+^ as a divalent cation cofactor improved the priming efficiency of blunt-ended or double-stranded oligodeoxynucleotides by 5–6-fold [75]. Meanwhile, the elongation rate of a hairpin substrate was increased ∼10-fold by optimizing the concentration of divalent cations [81]. Boulé *et al*. achieved the large-scale production of homogeneous TdT by lowering the growth temperature and overexpressing *argU* tRNA, which greatly benefited the biochemical and structural properties of TdTs [86]. The efficiency and processivity of primer extension are altered by the substitution of residues in the dNTP-binding pocket [63]. Mutations in the amino acids in the catalytic pocket, such as D395 N and E456 N in human TdT, result in equalized selectivity toward different dNTPs and changes in enzymatic activity [87].

Another big challenge is to enable TdT to incorporate 3′-blocked nucleotides, which is essential in DNA synthesis and modification. Ma et al. used I-TASSER and AlphaFold2 to predict the structures of TdT variants and performed two-site combinatorial mutagenesis to obtain an engineered ZaTdT that showed more than 1000-fold higher catalytic activity toward 3′-blocked-dNTPs than the commonly used MmTdT (*M. musculus* TdT) [80]. They have also focused on the development of rapid testing methods for screening TdT mutants with improved enzyme activity. Acoustic droplet ejection-open port interface-mass spectrometry (ADE-OPI-MS) was used to determine the TdT activity within 3 s for each sample. This high-throughput screening successfully identified several activity-enhanced mutants among approximately 10,000 TdT mutants, including the mutant ZaTdT-R335L-A193T-G337H—H478 G, which exhibited about a four-fold increase in catalytic activity for 3′-ONH_2_-dNTPs compared to the starting enzyme ZaTdT-R335L-K337 G [88]. Similarly, a combination of computational modelling and saturation mutagenesis in the GGFRR and TGSR motifs of TdT together with activity screening resulted in several functional mutants capable of utilizing 3′‑hydroxyl-blocked nucleotides (*e.g.*, 3′-*O*-amino, 3′-*O*-allyl or 3′-*O*-azidomethyl dNTPs) [89].

Furthermore, it is also important to enhance the thermostability of TdT to adapt it to various reaction conditions. To address this challenge, computational protein design has been applied to generate stable TdT variants [38]. Barthel et al. used the FireProt web server to predict 15 potential point mutations that could increase the thermostability of TdT and found that the optimal mutant MTdT-evo exhibited 2.3-fold higher activity for primers containing GC hairpins than the wild type enzyme at 47 °C [81]. Nirantar et al. adopted error-prone PCR to create a library of TdT mutants with an average of 2 to 5 amino acid mutations per gene and iteratively screened them for three rounds using a plate-based Förster resonance energy transfer assay [90]. Eventually, a bovine TdT variant TdT3–2 containing eight mutations with a Tm (the midpoint of unfolding transition for a protein) value 10 °C higher than the starting enzyme was obtained.

## 3 Applications of TdT

Owing to their unique ability to synthesize template-independent DNA, TdTs have attracted growing attention and have shown great application potential in many practical fields such as molecular biotechnology, biosensors, DNA microarrays, information storage, DNA synthesis, *etc.*

### 3.1 DNA construction and mutagenesis

**Fig. 5:**
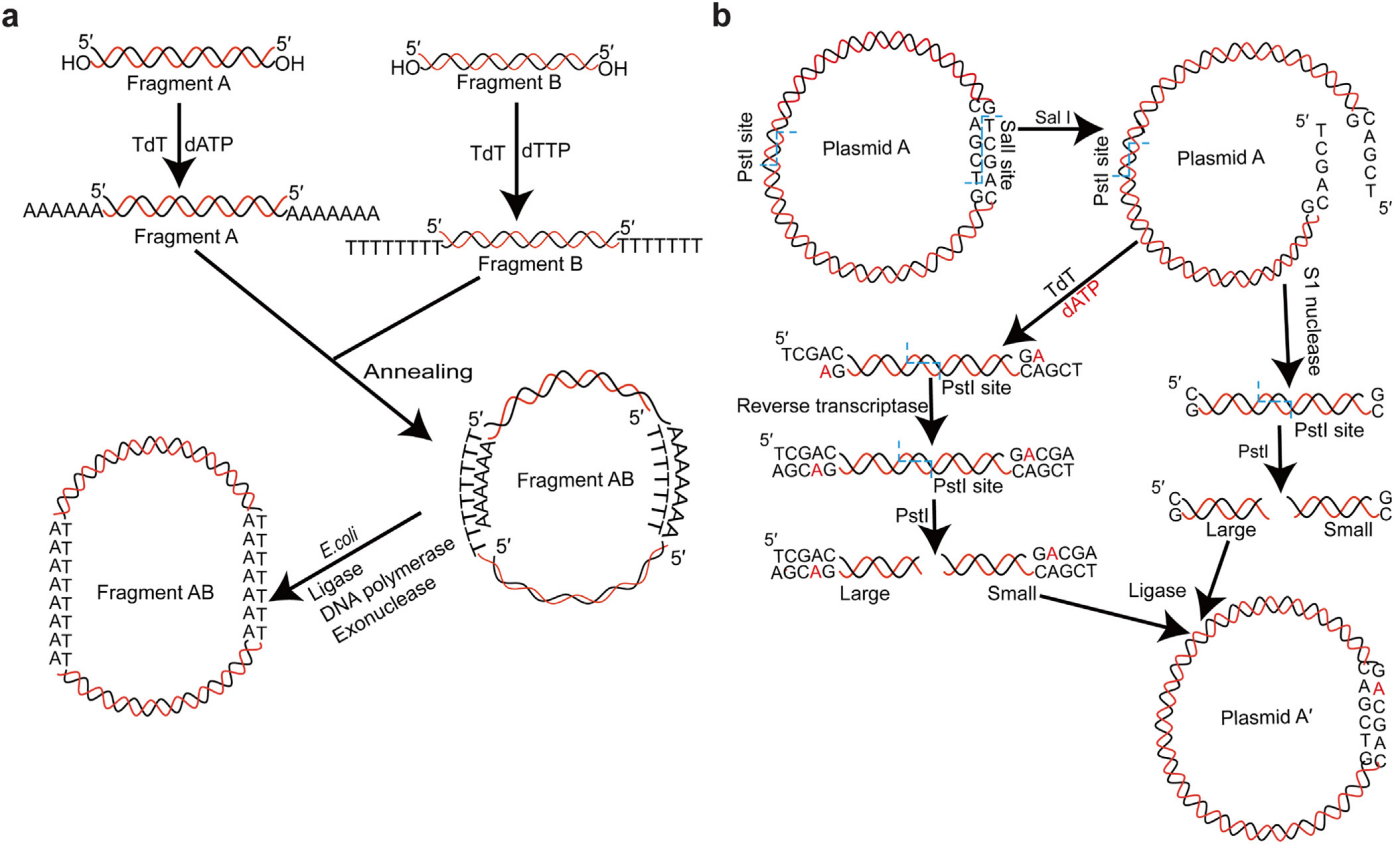
TdT-assisted DNA construction and mutagenesis. (a) DNA synthesis using TdT. TdT adds several dATP or dTTP nucleotides to the 3′ ends of two linearized duplex DNA strands. The two linearized DNA strands connect with each other through base-pairing annealing and are subsequently transferred to *E. coli* to form a complete circular DNA. (b) DNA mutagenesis using TdT. TdT adds a target base to the 3′ ends of restriction enzyme-cut plasmids. The elongated fragment fills the gap via reverse transcriptase, which lacks proofreading function, and therefore retains the mismatched base A. The unextended strand is processed by S1 nuclease to remove excess single strands. These two fragments are then cut by a different restriction enzyme and ligated by ligase to form a circular plasmid with a one-base mutation.

One of the earliest applications of TdT was the *in vitro* construction of recombinant DNA. TdT added homopolymers to the 3′-ends of duplex DNA, which enabled the covalent joining of any two DNA molecules [[91], [92], [93]]. Jackson et al. constructed a hybrid SV40 plasmid containing lambda phage genes and the galactose operon in *E. coli* (Fig. 5) [93]. This plasmid construction procedure involved four steps: adding homopolymer tails with specific composition to the 3′-end of a linearized plasmid or DNA fragment, adding complementary homopolymer tails to another DNA strands, annealing the two DNA fragments to form a circular DNA, and filling the gaps and sealing the nicks of the hybrid DNA to generate a covalently closed circular DNA [94,95]. The resulting hybrid DNA encoded most of the functions of SV40, *E. coli* galactose operon, and some phage λ genes. This has allowed the study of the molecular biology of SV40 and its interactions with mammalian cells. Thus, a bridge between the new non-viral genes and mammalian cells was established to further investigate their genetic and biological functions.

By incorporating tails to the 3′-end of a target DNA sequence, TdT was used to achieve *in vitro* mutagenesis, altering its expression and regulatory functions. Deng and Wu have described a useful procedure for generating mismatched nucleotides in duplex DNA [96]. Their *in vitro* method introduced a base change in only one DNA strand. When the altered plasmid was replicated in the host cells, one of the daughter plasmids became a mutant with a different base pair. Using this method, a specific base change was generated at the *Sal*I site of a DNA molecule.

### 3.2 Aptamer biosensors (aptasensors)

**Fig. 6:**
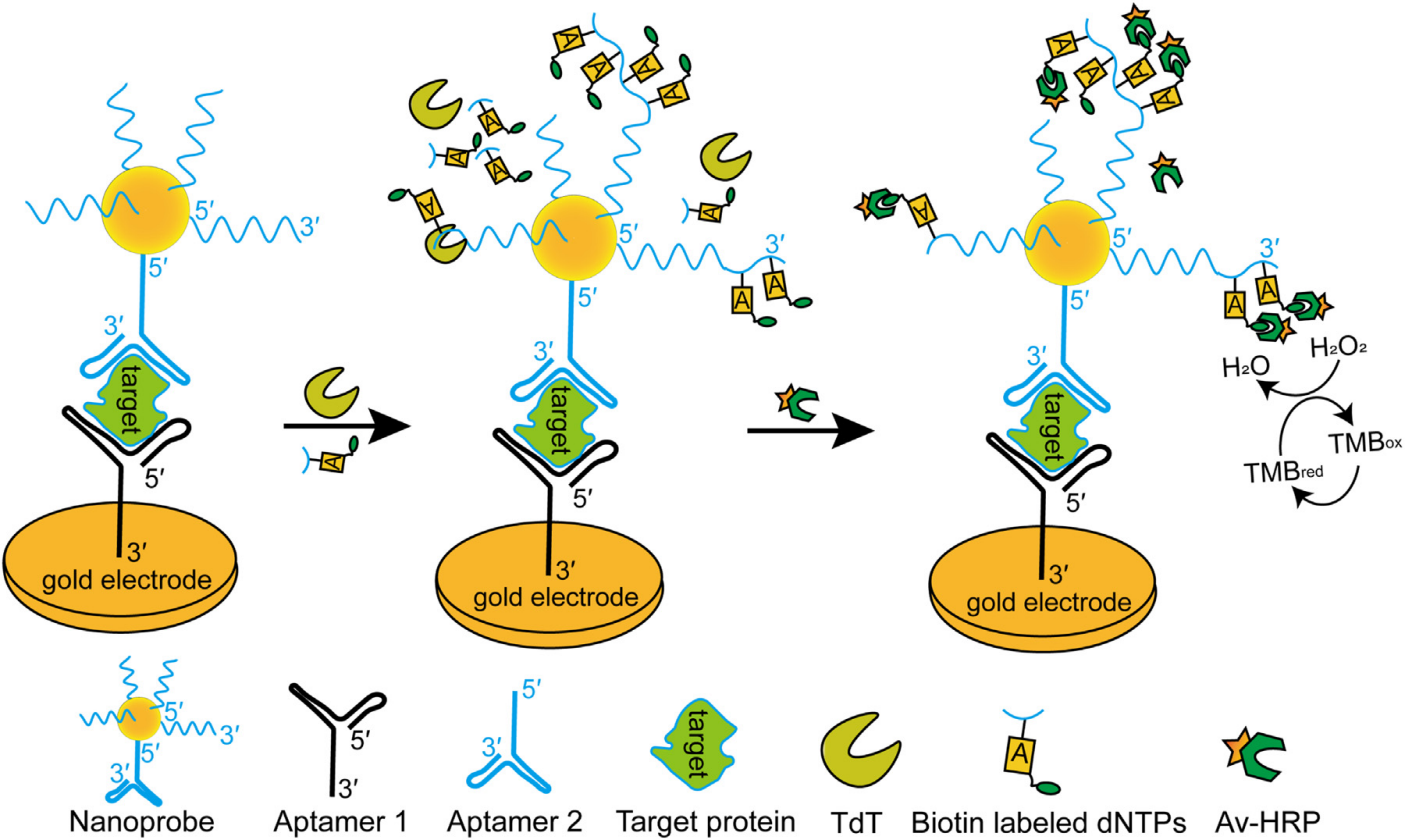
Use of TdT in the aptamer-OTEP strategy for electrochemical aptasensors. Aptamer 1 is attached onto the gold electrode via its 3′ terminus. Aptamer 2 is linked on the surface of Au nanoparticles via its 5′ terminus to form a nanoprobe. In the presence of the target protein, aptamer 1, the target protein, and the nanoprobe form a “sandwich” structure. TdT then catalyzes the elongation of aptamer 2 on the nanoprobe using biotin-labeled dNTP substrates, generating numerous long DNA strands on the Au nanoparticles that facilitate subsequent electrochemical signal amplification.

Owing to its ability to polymerize nucleotides, TdT can also be used as a part of a signal-amplified aptasensor for the detection of different diseases [[97], [98], [99]]. An aptamer-initiated on-particle template-independent enzymatic polymerization (aptamer-OTEP) strategy has been developed for early cancer diagnosis (Fig. 6). In this strategy, TdT extended the 3′-OH terminals of oligos on a nanoprobe with biotin labeled dNTPs. Then, avidin-modified horseradish peroxidase (Av-HRP) binds to the biotinylated strands and catalyzes the reduction of hydrogen peroxide to H_2_O, producing a sharp increase in the electrochemical signals. This method sensitively detected the cancer biomarker carcinoembryonic antigen in complex real samples with a detection limit of 50 fM and had a wider response range from the lowest detection limit of 5 fM to 500 nM compared to other reported electrochemical aptasensors. Considering these advantages, this strategy could be further improved and applied to the diagnosis of various cancers and other diseases, particularly when only small amounts of target protein samples are available [16].

### 3.3 DNA microarrays

**Fig. 7:**
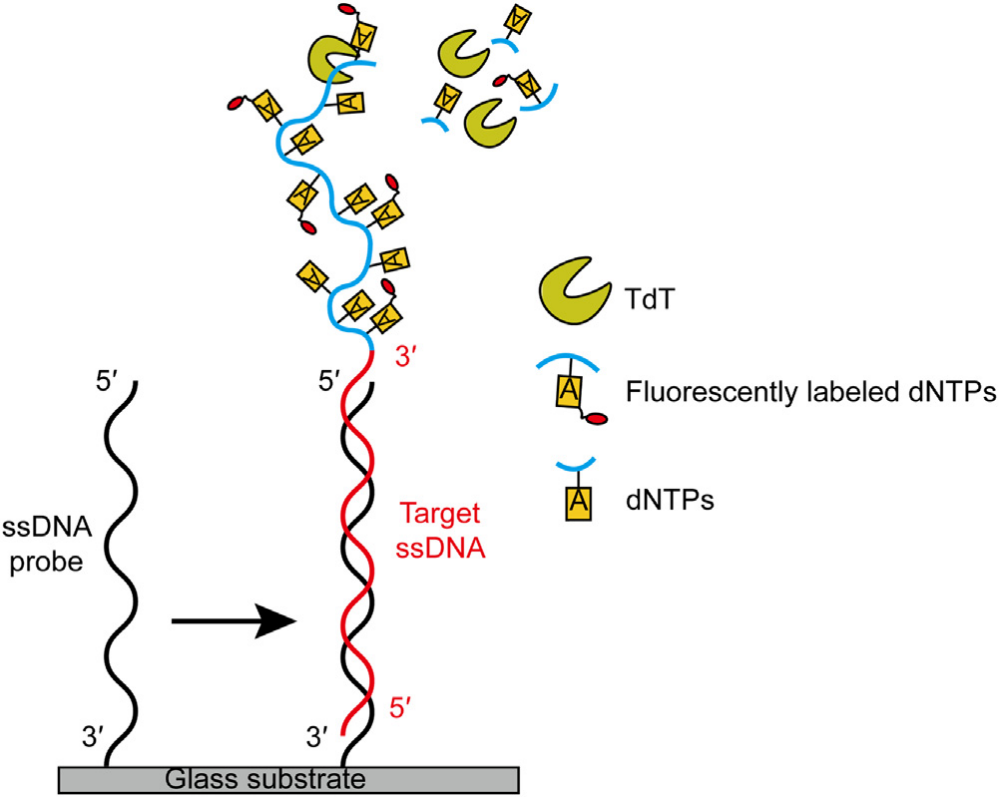
DNA fluorescence detection via surface-initiated enzymatic polymerization in DNA microarrays. A synthetic ssDNA probe is attached to the surface of the glass substrate via its 3′ end. When the target ssDNA complementary pairs with the immobilized ssDNA probe, TdT is activated to continuously add fluorescently labeled nucleotides to the 3′-OH ends of the target ssDNA, resulting in amplified fluorescence signals.

DNA microarrays are powerful tools in biology and medical research, involving applications such as the analysis of gene expression and the detection of microRNA [[100], [101], [102]]. TdT can also serve as a signal amplifier in DNA microarrays for nucleic acid analysis. Tjong et al. developed an isothermal, on-chip, post-hybridization labeling and amplification method called surface initiated enzymatic polymerization of DNA to detect target DNA or RNA molecules (Fig. 7) [103]. In this process, TdT integrates fluorescently labeled nucleotides into the 3′-OH ends of the target ssDNA on a microarray chip that contains strands complementary to the immobilized synthetic ssDNA probes. The fluorescent signal is then amplified, scanned, and analyzed. This approach could overcome some of the shortcomings of other widely used fluorescence-based arrays, most notably by avoiding complex front-end sample processing, which may add noise or complexity to the assay [101,104]. In addition, this method of signal amplification was performed under *in situ* and isothermal conditions, making it compatible with commercial microarrays. In addition, it generated a dose-response curve with a low detection limit of approximately 1 pM and a wide dynamic range of two orders of magnitude, making it a competitively sensitive method for nucleic acid determination assays.

### 3.4 Medical diagnostics

In the 1970s, TdT enzyme activity was proposed as a differential diagnostic tool for leukemia because it was only found in the thymus and bone marrow and its expression was elevated in some leukemias and lymphomas [19,105,106]. However, the TdT enzyme assay is limited by its difficulty in preparing fresh cells or cell extracts. Using the available bovine TdT antigen, Bollum provided an alternative method for developing an antibody for human diagnostic reagents, and detected new forms of TdT in human lymphoblastic leukemia and lymphoma [19,107]. This technique enabled the direct detection of TdT in clinical specimens at the cellular level. A more recent tool based on fluorescence signal amplification and TdT activity was developed for the sensitive detection of TdT. This approach successfully detected TdT activity in human serum with a detection limit of 0.093 U/mL, which could be exploited for the early detection and targeted therapy of leukemia [108].

**Fig. 8:**
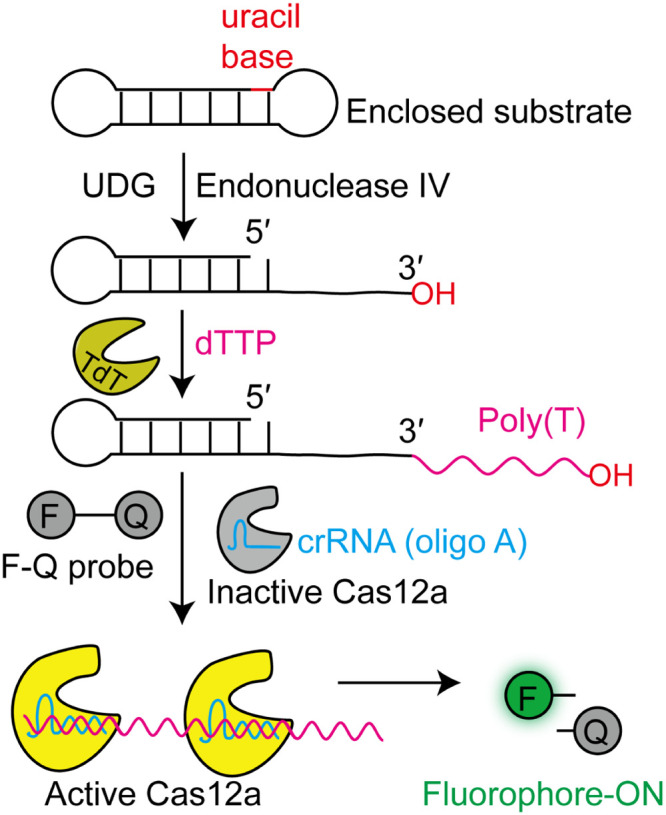
Detecting UDG activity using TdT and CRISPR-Cas12a. One uracil base in the stem of the enclosed substrate was removed using UDG to create an apyrimidinic site. Endo IV then cleaves the apyrimidinic site, releasing a free single strand with a 3′-end. Subsequently, TdT catalyzes the addition of dTTPs to form a poly(T) tail. The poly(T) tail pairs with the crRNA sequence (oligo A) to activate Cas12a and then mediates the cleavage of the F-Q probe to release the fluorescence signal.

Based on the target enzyme activity analysis, TdT is a useful tool for disease detection. Uracil-DNA glycosylase (UDG) is a vital enzyme in the DNA base repair process because it removes uracil, leaving an apyrimidinic site [109,110]. Abnormal UDG activity causes diseases such as immunodeficiency and cancer [111,112]. A simple and highly sensitive biosensing strategy combining TdT and CRISPR-Cas12a amplification was reported to achieve ultrasensitive detection of UDG activity in cancer cells (Fig. 8) [113]. This strategy adopted two amplification steps to obtain sufficient signal amplification. One involves TdT-mediated 3′-OH end extension of the substrate with dTTP to form a poly(T) sequence and the other involves the Cas12a-mediated cleavage of fluorophore/quencher pair-labeled ssDNA probe (F-Q probe). In this step, the crRNA of Cas12a was synthesized with a poly(A) sequence to recognize the poly(T) sequence and thus activate Cas12a to cleave the F-Q probe and release a fluorescent signal that could be detected by a fluorescence spectrometer. Therefore, it achieved zero background signals, a high signal-to-noise ratio, and a low detection limit of 5 × 10^–6^ U/mL in complex biological samples. This strategy was utilized not only to detect UDG activity in cancer cells, but also to screen UDG enzyme inhibitors, thus holding application potential in biomedical research and clinical diagnosis due to its cost-effectiveness and simplicity.

### 3.5 Detection and profiling of exosomes

**Fig. 9:**
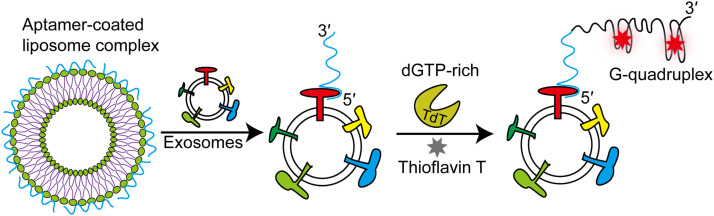
Detection and profiling of exosomes via TdT-mediated signal amplification. When exosomes are present, the aptamers detach from the liposomes and bind to them, exposing their free 3′- ends. TdT then elongates these aptamers with G-rich nucleotides to form G-quadruplex structures. Finally, G-quadruplex structures bind to thioflavin T to generate strong fluorescence signals.

Exosomes are extracellular vesicles that convey biological information between cells and act as biomarkers of pathological processes such as tumors [114,115]. Accordingly, the detection and characterization of exosomes is important for disease diagnostics [116,117]. An efficient and highly sensitive method for exosome detection has been developed based on aptamer-coated liposome complexes coupled with TdT-mediated G-rich polymerization (Fig. 9) [118]. DNA aptamers have a stronger and more specific binding ability to exosomes than to liposomes, owing to electrostatic interactions. When exosomes are present, aptamers bind to them rather than liposomes, thereby exposing their free 3′-OH. This triggers the polymerization of aptamers to form G-quadruplex structures and generate strong fluorescence signals, which reflect the amount of exosomal membrane protein proportional to the fluorescence intensity. As the entire reaction process was conducted in one pot, this method has the advantage of simultaneous mass sample analysis. Moreover, this method has great potential for the high-throughput analysis of various membrane proteins in different cancer cell-derived exosomes.

### 3.6 Information storage

**Fig. 10:**
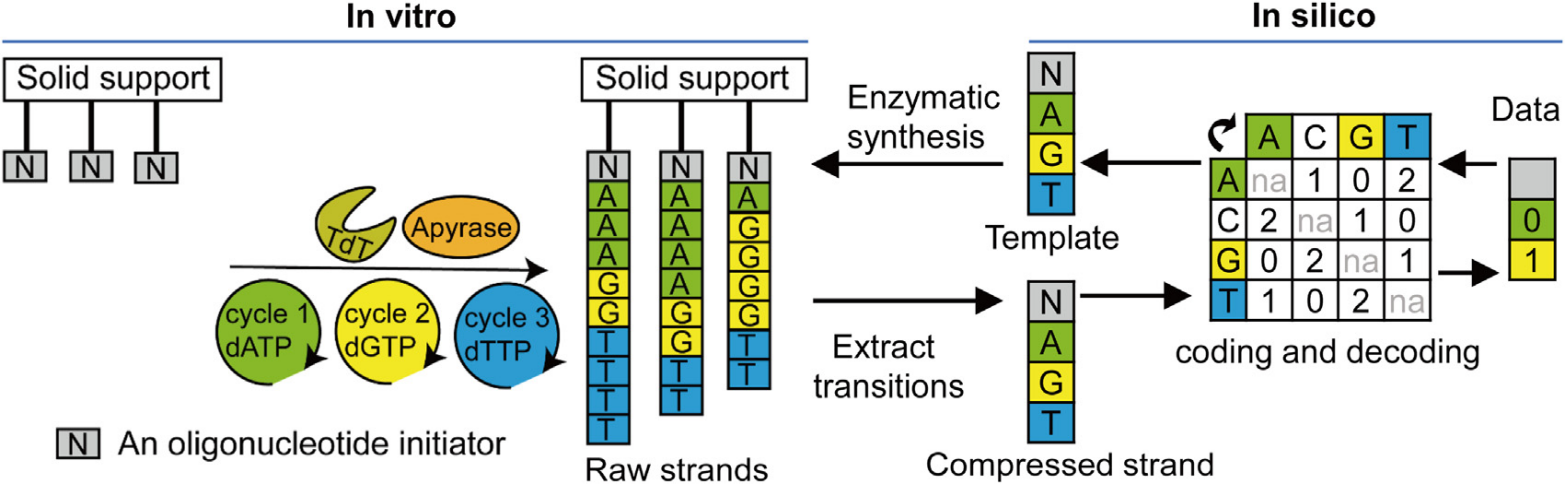
Enzymatic synthesis for DNA storage. Enzymatic DNA synthesis primarily involves the writing and reading of data. During the writing process, digital data are transformed into template nucleotides via *in silico* coding. Target nucleotide sequences are then enzymatically synthesized using TdT and stored *in vitro*. During the reading process, the stored DNA is sequenced and extracted into compressed DNA strands. Through *in silico* decoding, these DNA strands can be converted back into digital data.

DNA has many advantages as a medium for storing digital information, such as high density, energy efficiency, and long-term stability [119]. Therefore, digital DNA storage has attracted increasing attention in recent years [8,[120], [121], [122], [123], [124], [125], [126], [127]]. A novel strategy based on kinetically controlled *de novo* TdT-catalyzed synthesis with short homopolymeric elongation has been explored to enable digital information storage in DNA (Fig. 10) [128]. The TdT-catalyzed DNA synthesis system contains TdT, apyrase, and short oligonucleotide initiators. The reaction conditions and ratio of TdT to apyrase have been optimized to achieve a controlled number of nucleotide extensions for each cycle. Apyrase plays a critical role in competing with TdT by degrading its natural substrate dNTPs into TdT-inactive diphosphate and monophosphate forms. This ensures that each initiator in the synthesis cycle added at least one nucleoside triphosphate before the substrate was degraded by apyrase. Once the apyrase degraded all the added substrates, the next synthesis cycle began by introducing a different specific dNTP. Although the “extension length” (number of nucleoside triphosphates added) varied for each initiator in a cycle, the synthesized DNA strands all shared the same nucleotide transition (with one specific nucleoside triphosphate added per cycle). The resulting DNA sequences were then stored either *in vivo* within living organisms, such as bacteria, or *in vitro* as frozen powder.

When it became necessary to retrieve the stored data, the synthesized target sequences were read using Illumina sequencing technology. These sequences were converted into DNA and digital information to enable data recovery and use. In the proof-of-concept experiments of “hello world!” and “Eureka!,” storage efficiency rates of 1.5 bits and 1 bit per template nucleotide were obtained, respectively [128]. Although progress has been made in the enzymatic synthesis and digital information storage of DNA, this methodology still faces many challenges. For example, the storage capacity of DNA must be increased, and the corresponding hardware and automated fluid should be established to parallelize and manage massive amounts of digital data. Moreover, the digital codec might be tuned further to reduce the number of specific errors caused by G-quadruplexes in sequences rich in “G” nucleotides.

### 3.7 DNA synthesis

**Fig. 11:**
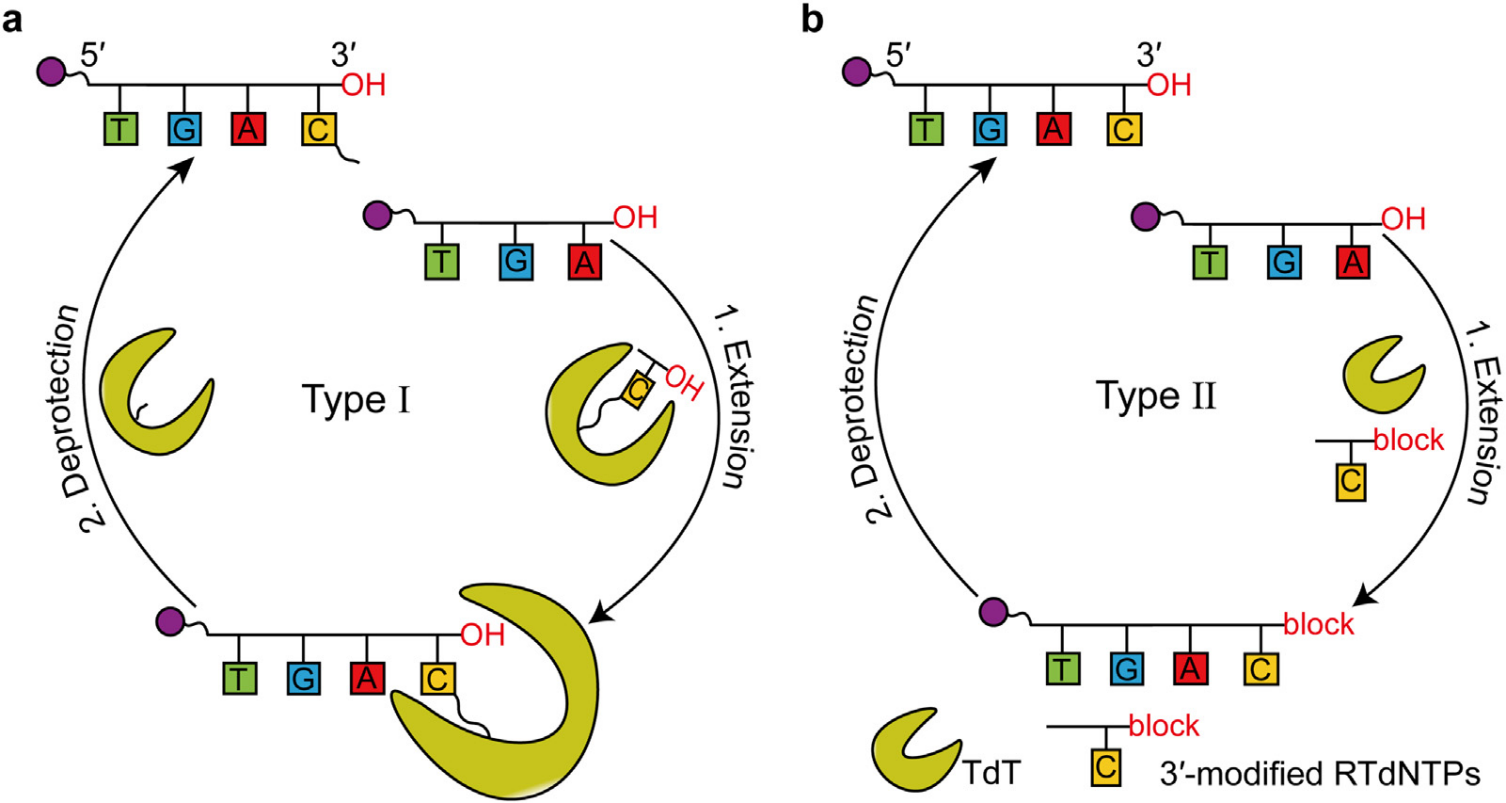
*De novo* enzymatic DNA synthesis strategies using TdT-dNTP conjugates and 3′-modified reversible terminator dNTPs (RTdNTPs). (a) The extension unit dNTPs are covalently attached to TdT enzymes (referred to as TdT–dNTP conjugates) via a cleavable linker connecting the base of the dNTP to the enzyme surface. After the extension step, the +1 product primers remain bound to the enzyme, thereby preventing the addition of the next substrate. (b) Extension unit dNTPs are protected at the 3′-OH position by reversible terminating groups, such as 3′-O-NH_2_. After the extension step, the +1 product primers retain their 3′-OH protection, making them unable to add the next RTdNTPs through the formation of 3′,5′-phosphodiester bonds.

Traditional DNA synthesis typically involves the use of nucleoside phosphoramidites via chemical methods. However, this approach has several drawbacks, such as generating hazardous waste and being limited to synthesizing only approximately 200–300 nucleotides (nt). In recent years, enzyme-based *de novo* DNA synthesis has emerged as a promising alternative strategy [14,[129], [130], [131]]. Based on TdT, two stepwise *de novo* DNA synthesis strategies (types I and II) were developed by adding one oligonucleotide to each extension cycle (Fig. 11). The overall strategy involved the synthesis of one nucleotide per cycle. Because the added nucleotides are 3′-blocked, the synthesized nucleotides cannot be elongated further, ensuring that only one nucleotide is extended per synthesis cycle. For example, to synthesize the DNA sequence “ATC,” three synthesis cycles are required: in the first cycle, only the mononucleotide “A” is added; in the second, only “T” is added; and in the third, only “C” is added. This way, the sequence “ATC” can be synthesized after three cycles.

One method (type Ⅰ) involves using of TdT-dNTP conjugates with 3′-unblocked dNTPs (Fig. 11a) [13]. Herein, TdT-dNTP conjugates are prepared using a short amine-to-thiol crosslinker between the accessible cysteine residue of TdT and the base of the dNTP analogs (propargylamino dNTPs). During extension, the DNA primer becomes incorporated into the TdT-dNTP conjugate. This results in the covalent attachment of the primer to the conjugates, preventing further extension due to steric hindrance. In the deprotection step, the extended primer is detached by cleaving the linkage between the incorporated nucleotide and TdT, for instance, by irradiation with a 405 nm laser for 1 min. This method successfully synthesized a 10-mer oligonucleotide with an average stepwise yield of 97.7%, performing well for repeating sequences like 5′-CCC-3′ [13,132].

The other method (type Ⅱ) employes 3′-modified reversible terminator dNTPs (RTdNTPs) (Fig. 11b), first suggested in 1962 [65]. In the extension step, the DNA primer incorporates a specific RTdNTP, such as 3′-*O*-(2-nitrobenzyl)- or 3′-*O*-NH_2_-dNTPs. The large 3′-modified removable group of RTdNTPs effectively blocks further elongation of the primer. In the deprotection step, physical or biochemical methods are used to remove the reversible groups, yielding a product primer with one desired nucleotide. A major challenge for this method is the elongation efficiency due to the limited space for 3′-OH modifications in the nucleotide binding pocket [65,89,133]. This method has successfully synthesized 10- or 50-mer oligonucleotides using engineered TdT and 3′-*O*-NH_2_-modified nucleotide substrates, achieving an average yield of up to 99%, higher than the TdT-dNTP conjugate method and comparable to traditional chemical synthesis (99.5%) [[134], [135], [136]].

Additionally, a theoretical model based on TdT was proposed for the synthesis of long DNA sequences in nanobioreactors [15]. This method adopts 3′-acetyl dNTPs (3′-Ac-dNTPs) as elongation units and deacetylase as the deblocking reagent. The feasibility of this approach was demonstrated through theoretical analysis based on available thermodynamic data and enzyme properties. However, limitations such as the undesired instability of TdT and 3′-Ac-dNTPs will remain a great challenge and need to be improved in future applications.

Leading companies in the field of DNA synthesis have developed various technologies and supporting instrumentation systems. For instance, DNA Script, a frontrunner in the manufacturing of *de novo* synthetic nucleic acids in France, released the first DNA printer called Syntax in 2020, based on enzymatic DNA synthesis technology [7]. This printer automatically synthesized 96 60-nt-long DNA oligonucleotides in parallel within 6–7 hours. Future generations of this technology are expected to synthesize DNA chains several hundreds or even thousands of nucleotides long. Moreover, DNA Script successfully synthesized a 280-base sequence using this enzymatic process, marking an important milestone. Another company, Camena Bioscience Ltd., developed a novel enzymatic *de novo* synthesis and gene assembly technology called gSynth™, which produced a series of 300 nucleotide fragments with an average accuracy of 85.3%, compared to 22.7% for traditional phosphoramidite synthesis (https://www.camenabio.com/). These advances inspired the synthesis of longer DNA fragments.

### 3.8 Metal ion detection

**Fig. 12:**
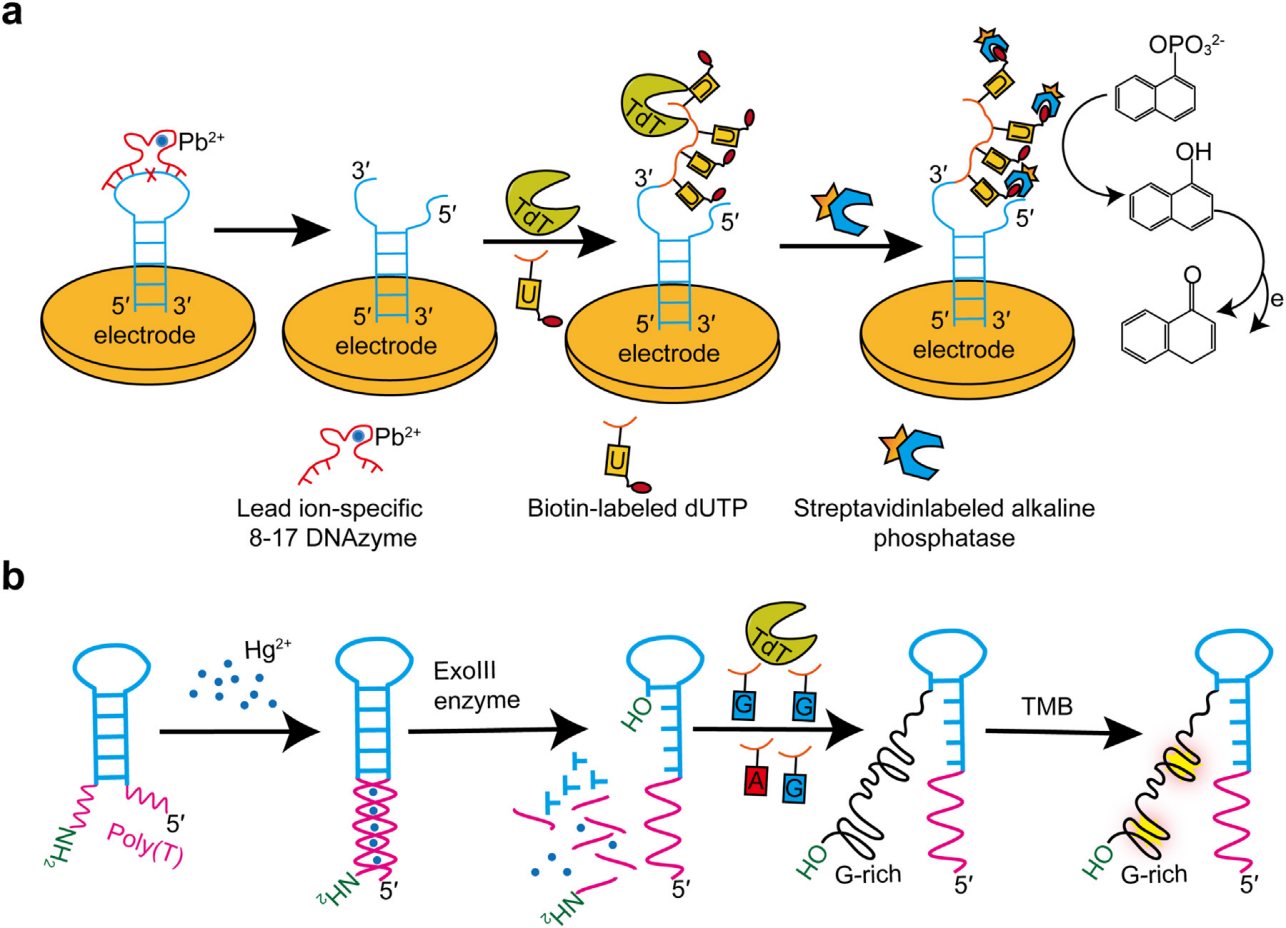
TdT-mediated detection of Pb^2+^ and Hg^2+^. (a) A Pb^2+^-dependent DNAzyme cleaves a hairpin-like substrate DNA strand, and then TdT adds biotinylated dUTPs to the released 3′-OH terminus. (b) The hairpin template sequences in the detection system form a blunt end with the help of Hg^2+^, which activate the catalytic activity of Exo III to cleave the 3′-end with -NH_2_. Thereafter, TdT adds G-rich nucleotides to its 3′- terminus to form G-quadruplexes.

TdT has also been applied to detect toxic metal ions such as lead and mercury ions [137,138]. Pb and Hg ions are harmful environmental pollutants that can severely damage human health even at low concentrations [139]. However, most existing methods for lead or mercury detection have drawbacks, such as the requirement of complex instruments and cumbersome preparation processes. Based on three cascades of amplification involving the Pb^2+^-dependent DNAzyme cleavage of hairpin-like substrate DNA strands, the incorporation of biotinylated dUTP to the released 3′-OH terminus by TdT, and the catalytic conversion of 1-naphthyl phosphate into active phenol for the generation of electrochemical signals, an electrochemical sensor was developed for the detection of Pb^2+^ ions (Fig. 12a). This method achieved a detection limit as low as 0.043 nM and excellent selectivity for Pb [138].

Similarly, an ultrasensitive and specific method based on exonuclease III (ExoIII) and TdT was developed to detect Hg^2+^ ions (Fig. 12b) [137]. The hairpin template sequences in the detection system form a blunt end in the presence of Hg^2+^, and the catalytic activity of ExoIII was activated to cleave the 3′-end with -NH_2_. TdT enzyme activity is then initiated to generate a large number of G-rich nucleic acid sequences. When incubated with iron (III)-hemin, these sequences exhibit peroxidase-like activity, catalyzing the oxidative coloration of 3,3′,5,5′-tetramethylbenzidine in the presence of H_2_O_2_. This system relies on mismatches between T-Hg^2+^-T and dual-enzyme amplification, making it highly specific and particularly sensitive, with a detection limit of 0.41 nmol/L.

## 4 Summary and outlook

The biological functions and catalytic mechanisms of TdT in the immune system have been investigated extensively. TdT is a critical participant in the V(D)J recombination process and confers a high diversity of antibodies and B-cell receptors. Moreover, the unique catalytic activity of TdT, adding natural or modified nucleotides to 3′-OH terminals of DNA/RNA or other chemical strands, endows itself with great research value and tremendous application potential.

Among the various applications discussed in this review, TdT-based *de novo* DNA synthesis appears to be the most promising. As an alternative to chemical synthesis, the screening and evolution of optimized TdTs and the development of corresponding devices for this green enzymatic strategy for DNA synthesis have broader prospects. To date, some laboratories and companies have successfully synthesized 10-mer or more than 200-mer DNA sequences. However, several unsatisfactory aspects, such as low extension yields, synthesis efficiency, short lengths of synthesized DNA strands, and high manufacturing costs, hinder its commercial availability. The critical part of the enzymatic method is obtaining the optimal TdT that exerts optimal activity and stability for all or separate target nucleotides. It is anticipated that, along with further studies on the synthesis of longer DNA strands, technologies on the enzymatic synthesis of DNA will mature and gradually replace chemical methods.

Many applications of TdT focus on detection-related fields, including the detection of enzymatic activity, mRNA, proteins, metal ions, and data storage applications. In most of these fields, detection approaches are based on the continuous incorporation of nucleotides to amplify signals. The detection limits of these techniques significantly improved with the assistance of the TdT enzyme. With gradual improvements in the enzymes used and rapid interdisciplinary developments in various fields, we envision that more successful and mature practical applications of TdT will emerge in the future.

## CRediT authorship contribution statement

**Chengjie Zhang:** Writing – review & editing, Writing – original draft. **Hizar Subthain:** Writing – review & editing. **Fei Guo:** Writing – review & editing. **Peng Fang:** Writing – review & editing. **Shanmin Zheng:** Writing – review & editing. **Mengzhe Shen:** Writing – review & editing. **Xianger Jiang:** Writing – review & editing. **Zhengquan Gao:** Writing – review & editing. **Chunxiao Meng:** Writing – review & editing. **Shengying Li:** Writing – review & editing, Supervision. **Lei Du:** Writing – review & editing, Supervision.

## Declaration of Competing Interest

The authors declare the following financial interests/personal relationships which may be considered as potential competing interests: Given his role as Executive Editor, Dr. Shengying Li had no involvement in the peer-review of this article, and had no access to information regarding its peer-review. Full responsibility for the editorial process for this article was delegated to Dr. Yoshizumi Ishino.
